# Medical certificate education: controlled study between lectures and flipped classroom

**DOI:** 10.1186/s12909-018-1351-7

**Published:** 2018-10-24

**Authors:** Nina Tusa, Erkko Sointu, Helena Kastarinen, Teemu Valtonen, Anna Kaasinen, Laura Hirsto, Markku Saarelainen, Kati Mäkitalo, Pekka Mäntyselkä

**Affiliations:** 1 Institute of Public Health and Clinical Nutrition, University of Eastern Finland, P.O. Box 1627, FI-70211 Kuopio, Finland; 2 School of Educational Sciences and Psychology, University of Eastern Finland, Joensuu, Finland; 3 Social Insurance Institution of Finland, Kuopio, Finland; 4 School of Applied Educational Sciences and Teacher Education, University of Eastern Finland, Joensuu, Finland; 5 Department of Applied Physics, University of Eastern Finland, Kuopio, Finland; 6 Faculty of Education, University of Oulu, Oulu, Finland; 7 Primary Health Care Unit, Kuopio University Hospital, Kuopio, Finland

**Keywords:** Flipped classroom, Lecture-based learning, Medical education, Medical certificate education, Quantitative research methods

## Abstract

**Background:**

Finnish permanent residents are covered by social security insurance administered by the Social Insurance Institution of Finland. The procedure of insurance is initiated with medical certificate written by the treating doctor. Thus, the doctor must have certificate writing skills accompanied with the knowledge of the content and goals for insurance. Quality certificates are important part of doctors’ professional skills worldwide and most effective teaching methods for learning these should be investigated.

**Methods:**

Medical certificate data were collected from two independent courses of fourth-year student taught in autumn 2015 (*N* = 141) and 2016 (*N* = 142) in the medical faculty of the University of Eastern Finland. A random sample of 40 students per course was drawn for the analysis. All certificates were analyzed as one sample. This was done to obtain reliable results with internal control group on the differences between two teaching methods, the traditional approach and the flipped classroom (FC) approach, in 2015 and 2016, respectively. The medical certificates were evaluated and scored with a rubric (range: − 4.00–14.25) by two independent experienced specialists.

**Results:**

Compared to students in the traditional classroom, students involved in the FC received significantly higher scores in all relevant sections of the assessed certificates. The mean of the total scores was 8.87 (*SD* = 1.70) for the traditional group and 10.97 (*SD* = 1.25) for the FC group. Based on the common language effect size, a randomly selected student from the FC group had an 85% probability of receiving a higher total score than a student from the traditional group.

**Conclusion:**

In this study, the FC approach resulted in a statistical significant improvement in the content and technical quality of the certificates. The results suggest that the FC approach can be applied in the teaching of medical certificate writing.

**Electronic supplementary material:**

The online version of this article (10.1186/s12909-018-1351-7) contains supplementary material, which is available to authorized users.

## Background

Medical education is facing new demands from twenty-first century society and work [1]. There has been a shift in medical education toward instruction methods that facilitate more active and student-centered learning [2, 3]. Lectures where students passively consume knowledge provided by lecturers are seen as ineffective and unresponsive to the demands for the skills that students need nowadays. For this shift, numerous activating teaching methods have been developed to facilitate students’ active role and involvement in learning situations [4–6].

One promising approach is the flipped classroom (FC) approach, which has several pedagogical roots in higher education [7]. The FC has been anticipated as one of the factors driving educational change in higher education and has been suggested for inclusion in mainstream education since 2014 [8, 9]. In recent years, the FC has also gained growing attention in medical education [10]. The main idea of the FC is to change the sequencing of instruction during courses [11]. Traditional lectures are replaced with pre-materials, typically online videos, which are studied individually before face-to-face (f-2-f) meetings. The f-2-f meetings are then devoted to active student-centered learning activities, allowing students to take advantage of the content expertise of their instructor for their learning needs. In a strict sense, the FC approach does not define the format or ways of using the pre-materials, nor does it define the pedagogical practices within f-2-f meetings. Typically, f-2-f meetings are designed using methods emphasizing collaborative [11–13] and/or student-centered, inquiry-based [14] learning practices that highlight students’ more self-regulative role in the learning process.

Previous studies on the FC have provided differing results. These studies have mainly focused on students’ experiences of the FC courses and learning results. The FC has been found to be a flexible [15] and enjoyable way of learning [16] leading to increased student satisfaction and interest with learning [6, 17]. The role of videos has been shown to be important in FC courses. Easy access to resources (e.g., videos) and own pacing of them in electronic platforms have been found to increase student satisfaction with teaching [18] and improve medical student’s self-learning abilities [17]. The use of (online) videos as part of courses has provided positive learning results [17, 19, 20]. FC methods with videos can provide an opportunity for students to better manage their cognitive load [21].

In their review of the medical education context, Chen, Lui, and Martinelli [10] reported that students have been satisfied with FC courses, particularly with the benefits of flexibility, easy access to resources, and more self-paced learning. The findings from studies focusing on learning results provide more variation. Examination results were significantly better when the FC model was used [22, 23], however, the learning results indicated no significant difference when the FC method and more traditional teaching and learning methods were compared [24, 25]. Based on a review study by O’Flaherty and colleagues [6], the overall learning results of FC courses were good in higher education and indicated increased academic performance. In the case of analyzing learning material, the FC approach may benefit student retention [26]. However, based on a review by Chen et al. [10] focusing on medical education, the results of FC courses have not been overtly positive. In line with these mixed results, Abeysekera and Dawson [21] considered the FC approach underresearched, underevaluated, and undertheorized in general and called for small-scale localized experimental studies to consider the efficacy of the FC model. Thus, the purpose of this study is to examine the FC within the context of medical education and medical certificate writing in Finland. More specifically, using an experimental research design, this study investigates differences in learning results between traditional teaching (i.e., lectures) and an FC with video-based lectures.

## Methods

### Study context

Undergraduate medical education in Finland is a continuous six-year program, where bachelor and master studies are integrated to form a licentiate degree. Medical education is based on a practical approach; for example, practical training with real patients frequently occurs as early as the first curriculum year. One important part of medical education is learning medical certificate writing skills.

In Finland, medical certificates are tied to the social security insurance system. Permanent residents are covered by insurance administered by the Social Insurance Institution of Finland (SII). The insurance covers income during sickness and reimburses medication expenses. The reimbursement rate depends on the disease and its severity, as well as the products confirmed as reimbursable by the National Pharmaceuticals Pricing Board.

To apply for entitlement to reimbursement of medication expenses at the special rate (≥ 65%), a medical certificate B issued by a treating doctor is needed. The treating doctor has a key role in initiating the insurance procedure, as he or she informs the patient and works as an expert. To fulfil this role, the doctor must know the content and goals of the applicable law. The entitlement to reimbursement is assessed and granted or denied by the SII based on this certificate. Therefore in this study, we considered it important to include an evaluator from the SII as the (future) doctor’s medical certificate B is evaluated by the SII and the reimbursement depends on the doctor’s skills.

### Participants

The data were collected from two independent courses taught in autumn 2015 (*N* = 141) and 2016 (*N* = 142) in the medical faculty of the University of Eastern Finland (UEF). The participants of the module were fourth-year students. The research was explained verbally to all students. Additionally, students received written information sheet that also included informed consent form. Participation to this study was voluntary, there were not any known risk factors for participating this study and data was planned to be protected according to the national/university policies. Written informed consent forms were received from 110 students from the 2015 course and 132 students from the 2016 course. Two students from the 2015 course and four from the 2016 course did not give us permission to use their responses in the study. Twenty-nine students from the 2015 course and six from the 2016 course were not reached out. This study follows the guidelines of Finnish National Board on Research Integrity (TENK) [27, 28]. According to these guidelines, participation should be voluntary, be based on informed consent, avoid any harm and collected data and participant privacy should be protected [27]. This study follows all of these aspects. According to the TENK guidelines [28], request for Ethical Review in Human Sciences should be addressed to the institutional review board (IRB) if: (1) physical integrity of subjects is violated, (2) principles of informed consent are not followed, (3) subjects are under 15-years old and the study is not part an institution’s normal activities, (4) research subjects are exposed to exceptionally strong stimuli and evaluation of possible harm requires special expertise, (5) long-term mental harm may be caused by the study, and (6) the study can increase the security risk of the subjects. Our study is human sciences research with medical education students, thus it is not research under the Medical Research Act (488/1999) [29]. This research used a quasi-experimental posttest-only design by using anonymised students reports. No backround data from students were drawn. Therefore, our study did not violate/contradict any of these above mentioned aspects (n:o 1–6) and is viewed as human sciences study. Thus, IRB statement was not needed in the current study according to the national guidelines.

From the two courses, a random sample of 40 students per course was drawn for the analysis. The sample was blinded for the evaluators independently by another researcher. Thus, the evaluators had no knowledge to which group students belong nor the identifying information of students. After the analysis, one student from the 2016 group had to be dropped from the sample, as the medical certificate B exercise was not finalized. Thus, the total sample size is 39 (50% female) for the 2016 course and 40 (53.8% female) for the 2015 course. The gender distribution and ethnic composition (< 5% of other than Finnish origins) represented courses of the previous years.

### Design and procedure

The study module is part of a bigger module called “Introduction to General Practice”. In 2015, the medical certificate writing was taught to students during a two-hour traditional lecture including nine other kinds of medical certificates (not reported here). In 2016, the instruction method was developed as part of the ongoing evidence-based development of learning environments at UEF. The instruction method of the 2016 course was modified to follow FC principles. Thus, the content of the traditional two-hour lecture was changed to short video-based FC lectures provided to students through an electronic learning platform. The duration of the video concerning medical certificate B investigated in this study (see Additional file 1) was 4 min and 39 s. In addition, nine other certificates were included in the video package, but they are not covered by the scope of this study. The same teacher taught this course in both years with traditional method and FC.

In both years based on the lectures and the same given material, every student did a medical certificate B exercise for a case patient. The patient had diabetes, and the purpose of the medical certificate B exercise was to apply for entitlement to reimbursement by SII of the diabetic medication expenses at the special rate (≥ 65%). The cases for both the 2015 and 2016 courses were presented in a similar manner for the students in electronic platform. In the instruction, the students were offered the criteria for the medical certificate B for diabetic medication. They were also told to investigate further information from the SII webpage as doctors do in real work settings. The purpose of the exercise was for the students to follow a real-life setting as closely as possible. In both years, the students also relied on peer support as they created small groups of three students to obtain feedback and correct their medical certificate B exercises. After this, each student individually returned the exercise to the teacher. Metadata from the media server right after the submission time indicated that the 2016 group had watched the medical certificate B video 263 times. At the end of the process, there were f-2-f sessions considering all the taught medical certificates.

The study used the assessment rubric for medical certificate B by the UEF Department of General Medicine (Additional file 2). The purpose of this rubric is to assess how students have learned to fill in the medical certificate B. The rubric follows the official medical certificate B sheet by the SII. The maximum total rubric score is 14.25. The minimum score can be negative, as negative scores can be given for sections 8–11. Markings in these sections are not needed in this task (the same medical certificate B sheet is also used for different purposes), and wrong diagnosis for the entitlement causes extra work for the SII. In addition to the total score, the rubric can be used to evaluate how students fill in information in the different parts of the medical certificate B: patient identification information, purpose of certificate (singe variable), background information (including medical history), examination findings, treatment plan, (diagnosis that justifies the) special refunds for medicines, doctor identifying information, and non-relevant information.

For the purpose of this research, the anonymized medical certificate B exercises were randomly combined as one 79-case assessment file by another researcher. Two specialized doctors (from SII and the university) read the medical certificates and assessed them independently with the rubric composed of 27 items per student. Discrepancies in the evaluations were found in five of the 79 cases. The doctors discussed these discrepancies and mutually agreed on the final scores of these five cases.

The research used a quasi-experimental posttest-only design with nonequivalent groups [30] from different cohorts in different years. The two samples from the same population (i.e., medical education students at the same university) consisted of a treatment group of students (2016; FC group), and a control group of students (2015; traditional teaching). In this case, the internal validity of the design was improved by using “internal controls” which tend to lead to fewer selection biases than using a control group from another university, for example, because the control group is more similar to the treatment group [30, 31]. SPSS v23 was used for the data analysis. We investigated the two groups’ data normality and found that the 2015 group data violated the data normality assumptions. Therefore, we chose to use non-parametric Mann-Whitney *U-*tests for the analysis to investigate the differences between groups. We also chose to report the means (*M*) and standard deviations (*SD*) to retain the original metrics of the rubric. The common language (CL) effect size [32] was used to estimate the probability that one sample had a higher score than the other sample.

## Results

Table 1 presents the range of rubric scores, the mean ranks (and *M* and *SD*) for the 2015 group (control) and 2016 group (treatment), the results of the comparison between groups (Mann-Whitney *U*), and the *CL* effect size. The 2016 students had a significantly higher total score (*p* < .01) as well as higher scores in the areas of patient identification information, background information, treatment plan, and special refunds for medicines, and a lower score in non-relevant information. The effect size (*CL* = 0.85) indicates that the total score of a randomly selected student from the 2016 group had an 85% probability of getting higher score than the total score of a randomly selected student from the 2015 group. For the specific measured areas, the probabilities that the score of a randomly selected student from the 2016 group would be higher than that of a student from the 2015 group were as follows: 68% for patient identification information (*CL* = 0.68), 73% for background information (*CL* = 0.73), 71% for treatment plan (*CL* = 0.71), and 69% for special refunds for medicines (*CL* = 0.69). Moreover, a randomly selected student from the 2016 group had an 82% probability of receiving a lower score than a student from the 2015 group in non-relevant information (*CL* = 0.82). The difference was significant (*p* < .05) in examination findings (*CL* = 0.63), where there was a 63% probability that a student in the 2016 group would receive a higher score than a student in the 2015 group.

**Table 1:** Results of the group 2015 and group 2016

	Range		2015 group		2016 group		Mann-Whitney U-test results			Effect size
	*min*	*max*	*Mean Ranks*	*M (SD)*	*Mean Ranks*	*M (SD)*	*U*	*z*	*p*	*CL*
Total Score	−4	14.25	26.03	8.87 (1.70)	54.33	10.97 (1.25)	221.00	−5.49	0.00	0.85
Patient identification information	0	3	33.93	2.59 (0.44)	46.23	2.83 (0.29)	537.00	−2.70	0.01	0.68
Background information	0	4.75	30.65	3.79 (0.78)	49.59	4.35 (0.52)	406.00	−3.76	0.00	0.73
Examination findings	0	1.5	35.39	0.96 (0.52)	44.73	1.21 (0.48)	595.50	−2.07	0.04	0.63
Treatment plan	0	1.5	33.96	1.04 (0.13)	46.19	1.19 (0.25)	538.50	−3.26	0.00	0.71
Special refunds for medicines	0	1.5	36.10	1.30 (0.41)	44.00	1.50 (0.00)	624.00	−2.93	0.00	0.69
Doctor identification information	0	1	43.25	0.73 (0.32)	36.67	0.62 (0.37)	650.00	−1.34	n.s.	n.s.
Non-relevant information	−4	0	28.15	−1.50 (0.85)	52.15	−0.54 (0.64)	306.00	−4.95	0.00	0.82

Note. *M* Mean, *SD* Standard Deviation, *CL* Common Language

No significant differences were found between the groups in the areas of doctor identifying information and purpose of the certificate. The mean ranks (and *M*) indicate that the 2016 group received higher scores in general for medical certificate B and a lower score in the non-relevant information scale. Wrong diagnosis can be an extra burden for the SII in an actual work context. Additionally, compared to the 2016 group, the 2015 group had a larger variation (as indicated by the *SD*) in all areas with significant differences, except in the treatment plan.

## Discussion

Medical certificates are commonly used around the world, but few, if any, studies focus on how to teach medical certificate writing. The purpose of this study was to investigate the FC approach within medical education and medical certificate writing in the Finnish context. It is essential to measure the effectiveness of different teaching methods to find the best methods to enhance the skills of medical doctors.

FC seems to be an effective method of teaching medical certificate writing. In practice, FC video lectures provide an opportunity to divide the teaching into practical parts. Students using the FC can benefit from its flexible way of studying; this flexibility can also lessen their cognitive load. In traditional lectures, students are tied to the schedule and to their note-taking abilities, whereas in the FC, learning is sequenced to allow the students to follow their own pacing. In this study, students received individual feedback from the teacher after they had tried their best in the medical certificate writing exercise, which most likely enhanced their learning experience. The structured feedback used in the study also makes the quality of evaluation consistent.

The FC method increases the possibility of learning. It seems to enable the medical students to learn the skills and knowledge needed in their future profession. The clarity of the FC videos and the possibility of revisiting these materials (the 2016 group watched the video 263 times) may further increase their motivation and self-efficacy for learning. Following Kirkpatrick’s [33] classification, this study aims to contribute to changes in knowledge and skills that influence the students’ future professional practice and patient outcomes.

The social security insurance administered by the SII has an essential role in reimbursing medical expenses in Finland. Doctors play a key role in the system. The medical certificate B sheet is also used for different, more complicated purposes, and teaching starts with the reimbursement case. Making a good medical certificate B demands extensive knowledge from the doctor. They must also master the social security insurance system and be able to understand the medical aspects of the patient.

### Limitations and future research

Although this study clarifies one aspect of medical education and increases the understanding of the FC method in medical education, it has several limitations. First, only one medical education institution and its students were included in the study. In the future, more comprehensive studies with randomized experimental design may increase the understanding of FC teaching. Second, the rubric used to evaluate students’ medical certificate B exercises was developed only recently; thus, there is no indication yet of the rubric’s psychometric properties. However, the study used a rigorous research design with a randomized sample and found only a few discrepancies between the evaluators’ assessments of the students’ medical certificate B exercises. Additionally, one of the doctors evaluating the students’ exercises was an official SII evaluator of certificates. These aspects increase the trustworthiness of the results. Nevertheless, in future studies, the psychometric aspects of the rubrics should be investigated. Third, the research design used only posttest data, limiting the possibilities to control the prior knowledge of the sample. Still, given the difficulty of getting into a medical education program and the fact that medical students are well educated in these programs, the variation between years can be seen as minimal and does not pose a threat to the study. Nevertheless, future research should include pretest data to its design. Fourth, these results consider only one type of medical certificate in one country; other types of certificates and countries should be included in future studies.

## Conclusion

In this study, the FC approach resulted in a statistical significant improvement in the content learning and technical quality of medical certificate writing. The results suggest that the FC approach can be applied in the teaching of medical certificate writing.

## Additional files

Additional file 1: Medical Certificate sheet. Official Medical Certificate B1 sheet used in Social Insurance Institution of Finland (SII). (PDF 1580 kb)

Additional file 2: Rubric for evaluating medical students’ medical certificate B exercises. Rubric scoring follows the Social Insurance Institution of Finland (KELA in Finnish) medical certificate B (Additional file 1). If the information was filled in correctly, the student received the designated weighted score; if not, a score of 0 was given. (DOCX 31 kb)

## Abbreviations

CL: common language
f-2-f: face to face
FC: flipped classroom
SII: Social Insurance Institution of Finland
UEF: University of Eastern Finland
