# Supplementary material for: Medical certificate education: controlled study between lectures and flipped classroom
Source: BMC Med Educ. 2018 Oct 24;18:243. doi: 10.1186/s12909-018-1351-7 (PMC6201511; doi:10.1186/s12909-018-1351-7)
Supplement: Supplementary file 1 — Medical Certificate sheet. Official Medical Certificate B1 sheet used in Social Insurance Institution of Finland (SII). (PDF 1580 kb) [file 12909_2018_1351_MOESM1_ESM.pdf]

Kela
Printed with the permission by KELA, i.e.,
Social Insurance Institution of Finland (SII).

Instructions for completing Medical Certificate B (SV 7e)

Depending on how extensive your statement is, you can select either the two-page B1 or the four-page B2 form. For a detailed assessment of working capacity, it is recommended to use the B2 form, which consists of two separate sheets.

2 PURPOSE OF THE CERTIFICATE
Tick the appropriate box. Rehabilitation subsidy corresponds to the earlier temporary disability pension.

3 HEALTH STATUS
List the diagnosed diseases affecting the examinee's capacity for work in order of significance (include ICD-10 codes).

4 BACKGROUND INFORMATION
Medical history, including the examinee's own assessment of his/her illnesses. Initial stage and development of the illness. Previous examinations and treatments. If the illnesses were diagnosed earlier, indicate the diagnostic methods used. Development of the examinee’s functional capacity and the effect of the illnesses, previous treatments and rehabilitation interventions. The examinee’s own account should be clearly distinguished from previous objective findings.

5 EXAMINATION FINDINGS
Objective examination findings, especially the examinee’s current clinical status. Focus on findings relevant to the purpose of the certificate. Where the purpose is to attest to the presence of a disease, list the findings most pertinent to the diagnosis and outline the reasoning behind the diagnosis. Where the purpose is to analyse the examinee’s capacity for work, describe the findings relevant to the examinee's performance capacity (e.g., a clinical stress test) and measurements of the examinee’s functional status under appropriate medication.

6 FUNCTIONAL STATUS
Provide an assessment, based on the examination findings, of the examinee’s functional status and any functional limitations imposed by the illness.

7 TREATMENT OR REHABILITATION PLAN
A documented treatment or rehabilitation plan is necessary to enable an assessment of the examinee’s work capacity and rehabilitation outlook. The goals and timetable set out in the plan are used to estimate the likely duration of incapacity for work.

Treatment plan. Treatment plan, including timetable, goals and expected results. Needed for the assessment of incapacity for work and for determining the examinee's entitlement to special refund of medicine costs.

Medical rehabilitation plan. Describe the rehabilitation plan or provide a proposal of the rehabilitation measures which should be carried out. Indicate the responsible rehabilitation providers. You may attach a separate document if you need more space to describe a detailed rehabilitation plan. If no rehabilitation is required, leave the space empty or, for the sake of clarity, indicate the reason why no rehabilitation is required.

Vocational rehabilitation plan. To be completed as needed. It is usually best to describe a detailed plan for vocational rehabilitation in a separate attached document, and to summarise its main points here. The need for vocational rehabilitation should be assessed from the standpoint of its potential for helping the examinee maintain or recover his/her working and functional ability. If vocational training or retraining is recommended, indicate the limitations imposed by the examinee’s health status in terms of his/her retraining prospects. Also identify the organisations or persons responsible or most suitable for drawing up the rehabilitation plan and for providing the rehabilitation services.

8 EVALUATION OF WORK CAPACITY
Type and extent of occupational activity. Needed for assessment of work capacity. Work history: previous employment relationships (include dates and job descriptions). If the examinee no longer works, indicate the date and reason for ending occupational activity. Describe the examinee’s current job and its physical and mental demands. How well is the examinee coping with his/her current job demands? Indicate the source of the information about the examinee’s job and occupational activity: whether obtained from the employer, an occupational health provider, or based on the examinee’s own account. Identify any occupational health provider involved for purposes of further information requests.

Work capacity. Provide an assessment of the examinee’s work capacity based on the health data, the assessment of functional capacity and the examinee’s occupational activity. If the examinee is assessed as being unfit for work, provide here, on the basis of the treatment or rehabilitation plan described above, an assessment of the prospects for restoring the examinee's work capacity and the estimated duration of incapacity. If the examinee is claiming partial sickness allowance, an occupational health physician familiar with the examinee's job assignments or other physician familiar with the examinee's working conditions must provide an assessment of whether the examinee would, while incapacitated for work, be able to perform some work without endangering his/her health or recovery.

9 STATEMENT BY THE OCCUPATIONAL HEALTH PROVIDER CONCERNING THE EXAMINEE'S ABILITY TO REMAIN IN WORK
The statement concerning the examinee's ability to remain in work should generally be made on Medical Certificate B if the purpose of the statement is also to offer an assessment of the duration of incapacity. If the purpose of the statement is solely to address the examinee's ability to remain in work, Medical Certificate A can be used as well. The sections to complete are 1, 5 and 7.

The statement by the occupational health physician must include an assessment of the examinee's remaining capacity for work and show the outcome of discussions with the examinee and his/her employer concerning the examinee's ability to remain in work. The statement must be made at the latest when sickness allowance has been paid for 90 working days. Both sections must be completed.

10 CONCLUSIONS
Tick the appropriate box and indicate relevant dates. In estimating the duration of work incapacity, refer to the information in the treatment or rehabilitation plan.

11 SPECIAL REFUNDS FOR MEDICINES AND CLINICAL NUTRIENTS
Indicate the diagnosis along with the ICD-10 code and a brief description of the treatment. More detailed information should be provided in the treatment plan. If you are proposing that the entitlement to special refunds should be temporary, indicate the date when the entitlement should end.

12 SIGNATURE
Physician's signature, stamp or printed name, identification number, speciality and place of work, and phone number.

Kela

Medical Certificate B1

concerning the examinee's health status, treatment/ rehabilitation and capacity for work

Number of documents enclosed: _________

Personal identity code

☐ Additional information on a separate sheet

1 PERSONAL DETAILS

Family name and given names | Telephone

Street address

Postal code | Postal district

Current job | Occupation

Principal employer

I have followed the examinee's health status

☐ personally since _____._____._________

☐ on the basis of documents since _____._____._________

How was the examinee's identity verified?

2 PURPOSE OF THE CERTIFICATE

☐ Sickness allowance

☐ Statement by the occupational health provider concerning the examinee's ability to remain in work

☐ Partial sickness allowance

☐ Other, please specify:

☐ Special refunds for medicines or clinical nutrients

☐ Rehabilitation

☐ Disability pension / Rehabilitation subsidy

3 HEALTH STATUS

List the diseases affecting the examinee's capacity for work in order of significance (including ICD-10 codes)

4 BACKGROUND INFORMATION

5 EXAMINATION FINDINGS

6 FUNCTIONAL STATUS

Background information. Initial stage and development of the illness. Previous examinations, treatment and rehabilitation and their outcome.

Examination findings. Clinical examination performed on _____._____._________ Weight _________ kg Height _________ cm
Tests of the examinee's functional capacity under appropriate medication; laboratory and imaging tests, and other tests (enclose findings).

Functional status. Based on the tests performed and your own professional judgment, indicate which activities of daily living the examinee is capable of. Describe any restrictions imposed by the examinee's health status.

Personal identity code

7<br>TREATMENT OR REHABILITATION PLAN	**Treatment plan** (e.g. medication, operative treatment, psychotherapy).<br>**Medical rehabilitation plan** (e.g. physiotherapy and occupational therapy, adaptation training, rehabilitation examination, inpatient rehabilitation).<br>**Vocational rehabilitation plan** (e.g. workplace adjustments, job try-out, work clinic or other rehabilitation examination, vocational training or job coaching) **or commission of a vocational rehabilitation plan.** Provide an assessment of the need and prospects of vocational rehabilitation. Name the institution or professional drawing up the plan or state your opinion as to who should draw it up. Participation of the examinee in the drawing up of the plan and the institution or professional carrying out the plan.<br>**Objective and schedule of the plans.**
8<br>EVALUATION OF WORK CAPACITY	**Please state what you know about the examinee's occupational activity and the type of work (s)he does.** Work history and description of current job. Level of physical and mental strain imposed by the job. Examinee's ability to cope with the demands of his/her current job.
	**Work capacity.** How does the impairment caused by illness affect the examinee's ability to cope with the demands of his/her current job or a comparable job? Describe the examinee's remaining work capacity. What effects do you expect the treatment and rehabilitation measures being proposed to have on the examinee's work capacity? When are the measures scheduled to be carried out? If you are not sufficiently acquainted with the examinee's occupational activity and its requirements, provide an estimate as to what types of occupational activity the examinee is capable of considering his/her remaining work capacity. If the examinee is claiming partial sickness allowance, an occupational health physician familiar with the examinee's job assignments or other physician familiar with the examinee's working conditions must provide an assessment of whether the examinee would, while incapacitated for work, be able to perform some work without endangering his/her health or recovery. Partial sickness allowance can be granted for a period of at least 12 and up to 120 working days.
9<br>STATEMENT BY THE OCCUPATIONAL HEALTH PROVIDER CONCERNING THE EXAMINEE'S ABILITY TO REMAIN IN WORK	Estimate of remaining work capacity
	Details of the examinee's ability to remain in work
	The statement by the occupational health physician must include an assessment of the examinee's remaining capacity for work and show the outcome of discussions with the examinee and his/her employer concerning the examinee's ability to remain in work. The statement must be made at the latest when sickness allowance has been paid for 90 working days. Both sections must be completed.
10<br>CONCLUSIONS	☐ I consider the examinee to be fit for work. ☐ I do not wish to comment on the examinee's work capacity.<br>☐ I consider the examinee to be unfit for his/her regular work or comparable work starting _____._____.________<br>☐ temporarily until _____._____.________ ☐ for the time being
11<br>SPECIAL REFUNDS FOR MEDICINES / CLINICAL NUTRIENTS	Disease ICD-10 code
	As it relates to treatment, this certificate is valid<br>☐ temporarily until _____._____.________ ☐ for the time being
12<br>SIGNATURE	**I declare that the above information is true and accurate.**<br>Date Physician's signature, stamp or printed name, identification number, speciality and place of work, and phone number
