# Supplementary material for: Medical certificate education: controlled study between lectures and flipped classroom
Source: BMC Med Educ. 2018 Oct 24;18:243. doi: 10.1186/s12909-018-1351-7 (PMC6201511; doi:10.1186/s12909-018-1351-7)
Supplement: Supplementary file 2 — Rubric for evaluating medical students’ medical certificate B exercises. Rubric scoring follows the Social Insurance Institution of Finland (KELA in Finnish) medical certificate B (Additional file 1). If the information was filled in correctly, the student received the designated weighted score; if not, a score of 0 was given. (DOCX 31 kb) [file 12909_2018_1351_MOESM2_ESM.docx]

| **Appendix 2**  *Rubric for evaluating medical students’ medical certificate B exercises* | | | |
| --- | --- | --- | --- |
| **Measured area** | **Explanation of the measured area** | **Weighted scoring** | **Section of Appendix 1** |
| Patient identification information | Personal identity code | 1.00 | top of page 2 |
|  | Family name and given names | 1.00 | 1 |
|  | Examinee’s health status follow-up time | 0.50 | 1 |
|  | Examinee’s identity verification | 0.50 | 1 |
|  |  |  |  |
| Purpose of the certificate | Marking the section on special refunds for medicines (or clinical nutrients) or explanation in sections 4–6 | 1.00 | 2 |
|  |  |  |  |
| Background information | Examinee’s age | 0.25 | 4 |
|  | Examinee’s gender | 0.25 | 4 |
|  | Examinee’s comorbidity | 0.25 | 4 |
|  | Examinee’s lifestyle | 0.25 | 4 |
|  | Hereditary risks | 0.25 | 4 |
|  | Manifestation of a disease | 1.00 | 4 |
|  | Diagnostic pathway | 0.50 | 4 |
|  | Laboratory tests justifying the diagnosis | 1.00 | 4 |
|  | Date of the laboratory tests | 1.00 | 4 |
|  |  |  |  |
| Examination findings | Height, weight, and BMI | 0.50 | 5 |
|  | Clinical examination | 1.00 | 5 |
|  |  |  |  |
| Treatment plan | Treatment plan | 1.00 | 7 |
|  | Aim of the treatment | 0.50 | 7 |
|  |  |  |  |
| Special refunds for medicines | Diagnosis to justify the reimbursement | 1.00 | 11 |
|  | Validity time | 0.50 | 11 |
|  |  |  |  |
| Doctor identification information | Doctor’s signature | 0.25 | 12 |
|  | Doctor’s identification number | 0.25 | 12 |
|  | Doctor’s working place | 0.25 | 12 |
|  | Doctor’s phone number | 0.25 | 12 |
|  |  |  |  |
| Non-relevant information | Evaluation of work capacity | -1.00 | 8 |
|  | Statement by occupational health provider | -1.00 | 9 |
|  | Conclusions for being fit to work | -1.00 | 10 |
|  | Special refund [wrong diagnosis] | -1.00 | 11 |
|  |  |  |  |
| Total score | All variables | 14.25 |  |

Note. Rubric scoring follows the Social Insurance Institution of Finland (KELA in Finnish) medical certificate B (Appendix 1). In the exercise, not all the sections needed to be filled in (only those important for the reimbursement). If the information was filled in correctly, the student received the designated weighted score; if not, a score of 0 was given. Additionally, negative scoring was possible in sections 8–11 for non-relevant information.
